# Comparative analysis of eight DNA extraction methods for molecular research in mealybugs

**DOI:** 10.1371/journal.pone.0226818

**Published:** 2019-12-31

**Authors:** Yu-Sheng Wang, Tian-Mei Dai, Hu Tian, Fang-Hao Wan, Gui-Fen Zhang

**Affiliations:** 1 State Key Laboratory for Biology of Plant Diseases and Insect Pests / Key Laboratory of Integrated Pest Management of Crop, Ministry of Agriculture and Rural Affairs of the People’s Republic of China, Institute of Plant Protection, Chinese Academy of Agricultural Sciences, Beijing, China; 2 Hunan Provincial Key Laboratory for Control of Forest Diseases and Pests, College of Forestry, Central South University of Forestry and Technology, Changsha, China; 3 Caofeidian Sub-Center of Hebei Entry-Exit Inspection and Quarantine Technical Center, Tangshan, China; 4 Center for Management of Invasive Alien Species, Ministry of Agriculture and Rural Affairs of the People’s Republic of China, Beijing, China; Swedish University of Agricultural Sciences, SWEDEN

## Abstract

For molecular research, the quality and integrity of DNA obtained will affect the reliability of subsequent results. Extracting quality DNA from scale insects, including mealybugs, can be difficult due to their small body size and waxy coating. In this study, we evaluate eight commonly used DNA extraction methods to determine their efficacy in PCR analysis across life stages and preservation times. We find that fresh samples, immediately upon collection or after 2 wks, resulted in the most effective DNA extraction. Methods using the DNeasy Blood & Tissue kit, NaCl, SDS-RNase A, and SDS isolated DNA of sufficient quality DNA. The SDS method gave high DNA yield, while the NaCl and SDS-RNase A methods gave lower yield. NaCl, SDS-RNase A, SDS, chloroform-isopentyl alcohol, and the salting-out methods all resulted in sufficient DNA for PCR, and performed equal to or better than that of the DNeasy Blood & Tissue kit. When time and cost per extraction were considered, the SDS method was most efficient, especially for later life stages of mealybug, regardless of preservation duration. DNA extracted from a single fresh sample of a female adult mealybug was adequate for more than 10,000 PCR reactions. For earlier stages, including the egg and 1st instar nymph samples, DNA was most effectively extracted by the Rapid method. Our results provide guidelines for the choice of effective DNA extraction method for mealybug or other small insects across different life stages and preservation status.

## Introduction

Mealybugs (Hemiptera: Pseudococcidae) are common pest insects that feed on the sap of a wide range of plants. Over 2000 species have been described from moist, warm climates globally, where they infest crops and ornamentals [1–3]. They can cause considerable yield and economic losses in invaded areas due to direct damage and disease spreading [4–7]. To prevent further spread and establishment of this and other invasive insect groups, techniques for early identification are required. Inspection and quarantine approaches typically intercept early life stages (eggs, nymphs) or residual debris, making morphological identification nearly impossible; only adults can be verified by morphology [8–10].

Molecular techniques are increasingly used for the detection and differentiation of insects, including taxonomy, genetics, and evolution, in addition to tracking invasions [11–14]. This type of molecular work requires DNA extraction from individuals [15–17]. Methods for DNA extraction vary in yield amount and quality [18]. The DNA extraction method employed should be appropriate for varied samples, maximize yield, and minimize contamination, degradation, and costs of money and time [14,18]. These ideal methods can be particularly difficult to establish when obtained specimens are very small or only a piece of an individual (*e*.*g*., a voucher specimen) can be used, making obtaining sufficient DNA difficult [16,19]. Mealybugs are small even as adults, with wax coatings on the body [20], and smaller life stages including eggs or newly emerged nymphs, or debris, are the samples most commonly found at quarantine inspection, compounding this problem.

DNA degradation in preserved specimens presents a challenge for DNA extraction and subsequent PCR amplification for large DNA fragments [21,22]. DNA condition typically depends on preservation method and on storage conditions [12,21–26], and DNA extraction methods [18]. Insect samples can be stored at room temperature, kept in solution, or cryopreserved [27–30]. Samples are commonly stored dry for long periods [25], which can result in degradation and loss of DNA [31]. Even after storage, certain reagents used for DNA extraction can limit the yield of DNA [26,32–34].

We tested the efficacy and reliability of eight common modern methods of DNA extraction from samples of mealybugs preserved for different durations of time. We assessed the yield and quality of DNA, as well as time and cost required for each method. PCR was used to assess the capacity for amplification and sequencing. Our results provide guidelines for which methods are most effective and efficient at procuring DNA for molecular studies of insects, particularly those samples that are very small and/or have been preserved for different lengths of time.

## Materials and methods

### Mealybugs and specimen preservation

Mealybugs were collected in the field or reared in our laboratory. The field collected specimens of closely related mealybug species were preserved over different time periods. In total, four time periods of specimen preservation were included, including fresh (0 wk), short (2 wks), intermediate (72 wks), and long (137 wks) (Table 1). The specimens were deposited in the Center for Management of Invasive Alien Species, Ministry of Agriculture and Rural Affairs of the People’s Republic of China, Beijing, China.

**Table 1:** Mealybug specimens preserved at different time periods for DNA extraction.

Code	Status of specimens	Period (wks)	Species	Origin	Location
I	Fresh (living specimen)	0	*Planococcus citri*	Lab rearing	Langfang (39°30'32.64" N, 116°36'29.03" E)
II	Short period conserved (-80 °C with 99.7% ethanol)	2	*Phenacoccus solenopsis*	Field collection	Haikou (20°3'22.50" N, 110°19'54.12" E)
III	Intermediate period conserved (lab condition with 99.7% ethanol)	72	*Phenacoccus solani*	Field collection	Kunming (25°7'41.32" N, 102°44'54.38" E)
IV	Long period conserved (lab condition without any medium, dry specimen)	137	*Dysmicoccus neobrevipes*	Field collection	Kunming (25°2'18.39" N, 102°39'53.26" E)

No specific permissions were required for the described field studies. (a) No specific permissions were required for these locations/activities because the mealybugs are pests on common crops or from lab rearing; (b) The locations are not privately-owned in any way; (c) The field studies did not involve endangered or protected species.

### Methods for DNA extraction

Eight common modern DNA extraction methods were employed in this study, including:

Method 1 (M1), the sodium chloride method (NaCl), as described by Shi *et al*. [35]. Salt solution (5 M NaCl) was used to remove cellular protein and concentrate genomic DNA. Isopropanol was used for desalting and DNA precipitation, and RNase A was used to remove contaminant RNA.
Method 2 (M2), the sodium dodecyl sulfate (SDS)-RNase A method (SDSR), as described by Phillips & Simon [36]. The 5 M NaCl and isopropanol were used as in M1. To remove contaminant RNA, RNase A was used.
Method 3 (M3), the sodium dodecyl sulfate method (SDS), as described by Phillips & Simon [36]. Procedures were exactly consistent with M2, but the RNase A was not used.
Method 4 (M4), the DNeasy Blood & Tissue kit (DNeasy) (Qiagen, Duesseldorf, Germany). The kit combines the binding properties of a silica-based membrane with simple microspin technology.
Method 5 (M5), the chloroform-isopentyl alcohol method (Chloroform), as described by Zhou *et al*. [37]. Chloroform/isopentyl alcohol (v:v = 24:1) was used to remove protein and concentrate DNA, and ethanol was used for desalting and DNA precipitation.
Method 6 (M6), the acetic acid potassium method (KAc), as described by Dai *et al*. [38]. Salt solution (3 M KAc) was used to remove protein and concentrate DNA and ethanol was used as in M5.
Method 7 (M7), the salting-out method (Salt), as described in Sunnucks & Hales [39]. The 5 M NaCl and ethanol were used as in M1 and M5.
Method 8 (M8), the rapid method (Rapid), as described in De Barro & Driver [40]. This method is a crude DNA extraction method; DNA could be used for PCR amplification directly without precipitation or dissolution.

The detailed procedures of each method are described in S1 File.

### DNA extraction

For each DNA extraction method, total DNA was individually extracted from 20 mealybugs, *i*.*e*., one individual of each ontogenic stage (including egg, 1st and 2nd instar nymphs, 3rd instar female nymph, and female adult) of mealybug specimen preserved for four durations was included. To remove the wax powder coating on the body surface, mealybug specimens (except egg and 1st instar nymph) were soaked in trichloromethane for 30 min, and then kept in 99.7% ethanol until further use. Prior to DNA extraction, the specimens were soaked in ultrapure water for 6–10 h and then naturally air-dried on a piece of sterile filter paper. The pretreated mealybugs were individually homogenized on a piece of parafilm (Bemis, Neenah, WI, USA) using a sterile PCR tube (as the pestle) with 20 μL DNA extraction buffer. The homogenate was placed in a 1.5 mL centrifuge tube. The homogenizer (*i*.*e*., the parafilm and the PCR tube) was washed twice with DNA extraction buffer, then transferred to the same centrifuge tube. Ultrapure water (20 μL for egg, 30 μL for 1st and 2nd instar nymphs, and 40 μL for 3rd instar nymph and female adult) was used to dissolute the extracted DNA. The detailed procedures are described in S1 File.

### Electrophoresis analysis of extracted DNA

To ensure DNA quality, 1 μL of the resuspended DNA was directly visualized on 0.8% agarose gel at 110 V for 30 min. The electrophoretogram was observed by using the GelDoc XR^+^ Imaging System (Bio-Rad, Hercules, CA, USA).

### DNA absorbance ratio and yield rate

Due to the small amount of DNA extracted from a single egg or a 1st or 2nd instar nymph, the DNA concentration and absorbance ratio could not be measured accurately in these samples by using spectrophotometry. The DNA concentration (S1 Table) and absorbance ratio (A260/A280) (S2 Table) of 3rd instar nymph and female adult were measured individually by spectrophotometry (NanoPhotometerP330, Implen, Munich, Germany). We measured air-dried body mass by using electronic analytical balances (XS105 DualRange; Mettler-Toledo, Zurich, Switzerland) in groups of nine or 10 individuals for the 3rd instar female nymph and female adult, because the mealybugs are tiny insects, with a female adult size of about 2.9 × 1.3 mm (the nymphs are even smaller, S3 Table) and difficult to measure body mass individually. Totally, three groups were measured, and individual body mass of 3rd instar female nymph and female adult was estimated accordingly (S4 Table).

To compare the efficiency of the eight DNA extraction methods, average DNA yield rates (S5 Table) (DNA ng per mg of body mass, considering the various body size of different mealybugs and different life stages) were calculated and compared based on the DNA concentration (S1 Table), DNA volume, and body mass (S4 Table). No significant differences in the states of DNA between 3rd instar female nymph and female adult or among closely related mealybug species [18,21], and because the DNA concentration of some specimens preserved for intermediate and long periods are inaccurate or even unmeasurable (S1 Table) since DNA degradation after longer storage [26], four individual mealybugs (fresh or short period preserved samples: one 3rd instar nymph and one adult, respectively) were included as four repeats. And then, DNA yield rate (log-transformed for normal distribution) and absorbance ratio were analyzed using one-way ANOVA followed by Tukey’s HSD test with a threshold of P < 0.05 in SPSS Statistics version 19.0.0 (SPSS Inc, Chicago, IL, USA).

### PCR amplification of genomic DNA

To assess DNA quality for PCR amplification, mitochondrial cytochrome c oxidase (mtDNA COI) and nuclear DNA 28S ribosomal DNA (28S rDNA) were amplified from each DNA sample. The universal barcoding primer pair PcoF1 (5’-CCTTCAACTAATCATAAAAATATYAG-3’) and LepR1 (5’-TAAACTTCTGGATGTCCAAAAAATCA-3’) [41] was used to amplify a fragment (~710 bp) of the COI gene. The fragment of the 28S gene (~780 bp) was assayed by using the primer pair 28SF (5’-AGTCGKGTTGCTTGAKAGTGCAG-3’) and 28SR (5’-TTCGGGTCCC AACGTGTACG-3’) [42].

Each 25 μL PCR reaction mix contained 2.5 μL of 10 × reaction buffer, 1.5 μL of DNA template (unnormalized DNA concentrations), 0.4 μL of dNTPs (10 mM), 0.4 μL of TransStart *Taq* DNA polymerase (2.5 U·μL^-1^; TransGen Biotech Co., Ltd, Beijing, China), 0.4 μL of each primer (10 μM), and 19.4 μL of ultrapure water. For amplification of the COI gene, the PCR thermal regime consisted of 2 min at 94 °C, five cycles of 40 s at 94 °C, 50 s at 45 °C and 50 s at 72 °C, 35 cycles of 40 s at 94 °C, 50 s at 51 °C and 50 s at 72 °C, and 5 min at 72 °C. The 28S gene was amplified under the following conditions: 2 min at 94 °C, 35 cycles of 40 s at 94 °C, 50 s at 58 °C, 1 min at 72 °C, and 5 min at 72 °C. All PCR products were resolved in 1.0% agarose gel at 100 V for 30 min.

### Time and cost estimation

The period of time required to finish one extraction from an individual mealybug using each of the eight methods was estimated, excluding the steps of solution preparation and pretreatment of the mealybugs. The cost of one extraction for each method was calculated based on the cost of DNA extraction kits, chemical reagents, enzymes, and disposable items (*e*.*g*., centrifuge tubes, parafilm, pipette tips). Combined cost and time were calculated as: (estimated cost per extraction of any one method / maximum estimated cost among eight methods) × (estimated time per extraction of any one method / maximum estimated time among eight methods).

### Application of DNA extracted with the SDS method

Based on the above comprehensive analysis, the SDS method (M3) was generalized as the most suitable for DNA extraction. To evaluate the quantity of available DNA extracted by M3, PCR amplification of COI and 28S genes was tested on 2-fold serial dilutions of DNA extracted from fresh samples (Preservation status I) of individual mealybug female adults. Concentrations of the DNA serial dilutions ranged from 166.20 (±5.40) × 10^3^ pg·μL^-1^ to 10.14 pg·μL^-1^ (1- to 16,384- fold dilution of the original extracted DNA). PCR assays were performed on five individuals. PCR conditions were the same as described above. PCR products were resolved in 1.0% agarose gel, and bidirectionally sequenced by Beijing Sunbiotech Co., Ltd. (Beijing, China). A BLAST search was conducted to confirm whether the sequences generated in the present study were part of the fragments of the COI or 28S gene.

## Results

### Qualitative evaluation

#### Electrophoresis of extracted DNA

**Fig 1:**
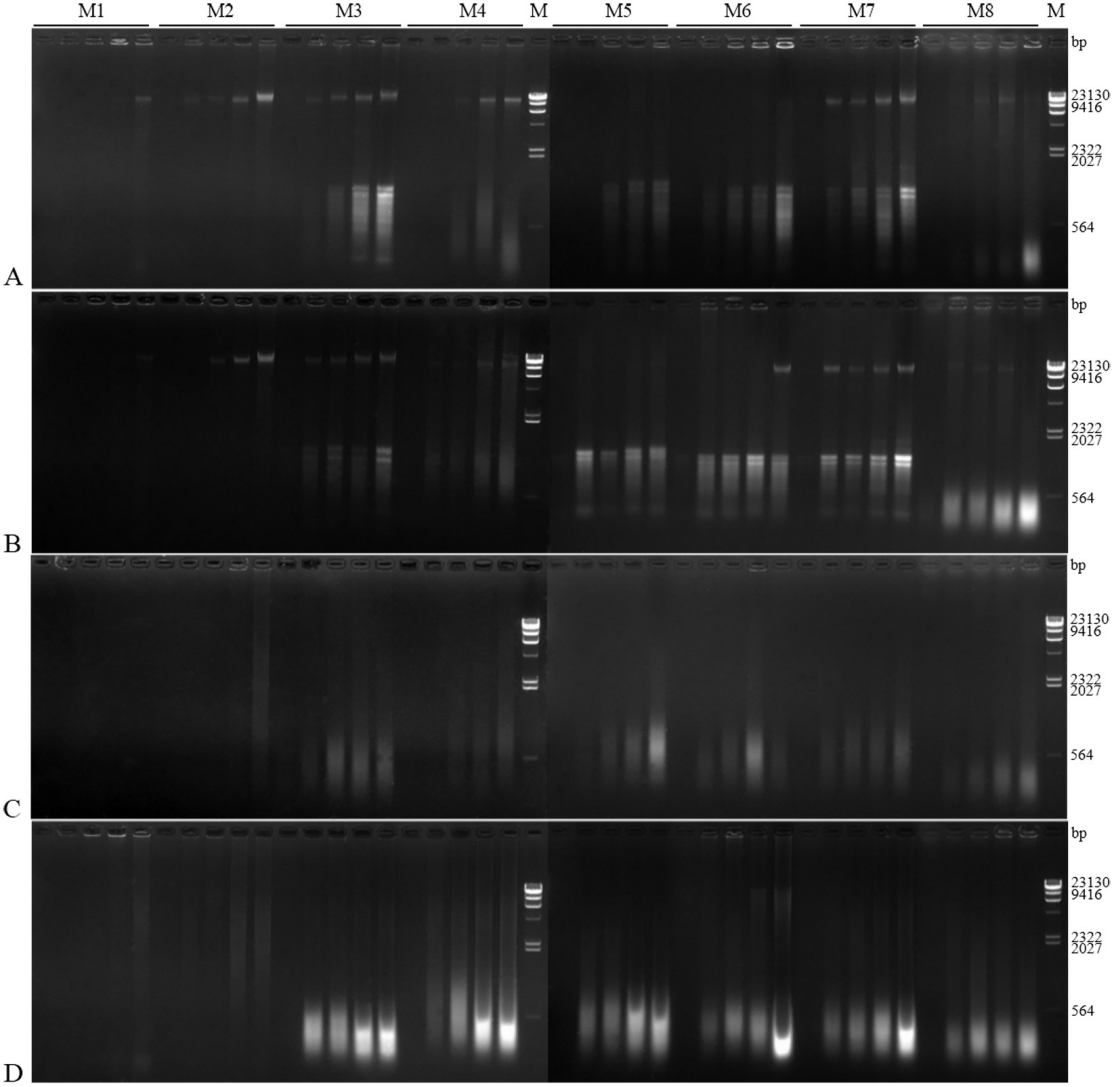
Electrophoresis of total DNA extracted from specimens preserved for four durations by using eight commonly used methods. (A-D) DNA extracted from specimens preserved for different durations (A: fresh, B: short period, C: intermediate period, D: long period; coded as I-IV in Table 1). Five lanes are shown for each method, from left to right: DNA samples extracted from an individual egg, and 1st, 2nd, and 3rd instar nymphs, as well as female adult mealybugs. M: λ-*Hind* III digest DNA marker.

Detection by electrophoresis indicated that the fresh and short period preserved samples (Preservation status I and II) (Table 1) resulted in the best quality of DNA (Fig 1). The length of the major DNA fragments was about 23 kb, showing that the extracted genomic DNA was intact with minimal degradation [26]. DNA bands obtained by using the methods of SDSR (M2), SDS (M3), and Salt (M7) were brighter than those obtained using DNeasy (M4) (Fig 1A and 1B). DNA extracted by using the M3 and M7 methods presented smear tails, indicating that a small amount of DNA had been degraded or the DNA was contaminated with RNA [43] (Fig 1A and 1B). The NaCl (M1) and M2 methods, using RNase A to remove RNA, obtained high quality DNA with the absence of a smear tail. The amount of DNA extracted by using M2 was greater than that obtained by using M1 (Fig 1A and 1B). Major visible DNA bands were detected using the Rapid (M8) method with specimens of 1st, 2nd, and 3rd instar nymphs, while less success was observed with the DNA extracted from female adults (Fig 1A and 1B). Chloroform (M5) and KAc (M6) performed the worst for DNA extraction, with the major visible DNA band almost undetectable (Fig 1A and 1B).

In samples from specimens preserved over medium and long periods (Preservation status III and IV) (Table 1), no band was detected in DNA extracted from any ontogenic stage with the methods M1 and M2, nor from eggs with the other six methods (Fig 1C and 1D). DNA extracted from the 1st, 2nd, and 3rd instar nymphs and female adults with the other six methods only gave smears with a molecular weight lower than 1,000 bp (Fig 1C and 1D), showing that the DNA had been sheared or degraded.

#### PCR amplification

**Fig 2:**
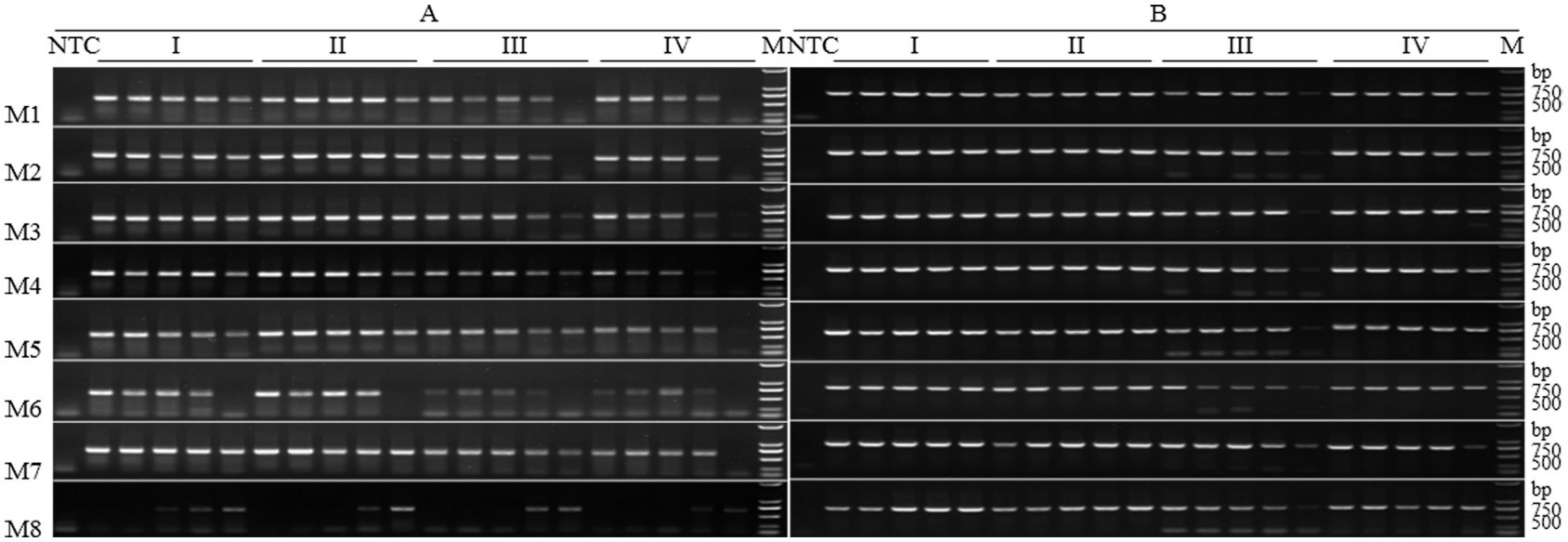
PCR amplification pattern of COI (A) and 28S (B) genes for testing the DNA quality extracted specimens preserved for four durations by using eight commonly used DNA extraction methods. Preservation methods I-IV are fresh, short period, intermediate period, and long period. Five lanes are shown for each method, from left to right: DNA samples extracted from an adult mealybug, 3rd, 2nd and 1st instar nymphs, as well as individual eggs. M: Trans2K DNA Marker; NTC: negative control (ultrapure water).

To assess the DNA quality, PCR reactions were performed on each DNA sample. The ~710 bp fragment of the COI gene was successfully amplified from DNA samples extracted by M3 and M5 (Fig 2A). Amplification was consistent, even for eggs, which have the smallest size and lowest DNA quantity. Specimens preserved for the intermediate period (Preservation status III) were consistent, but a weak band was observed for eggs preserved for the long period (Preservation status IV). The partial fragment of the COI gene was successfully amplified from more than 90% of the DNA samples (18/19 vs. 20) extracted with the methods M1, M2, M4, and M7 (Fig 2A), with the exception of eggs preserved for intermediate and long periods. DNA extracted from eggs and 1st instar nymphs by using the method M8 was detectable (Fig 2A), whereas DNA extraction using M6 had a low success rate (Fig 2A).

To further evaluate the quality of the extracted DNA, another gene, the 28S rRNA, was amplified. A ~780 bp fragment of 28S was successfully amplified from all 20 DNA samples extracted by using any of the eight methods (Fig 2B), indicating that the isolated DNA was high quality.

### Quantitative evaluation

#### DNA absorbance ratio and yield rate

**Fig 3:**
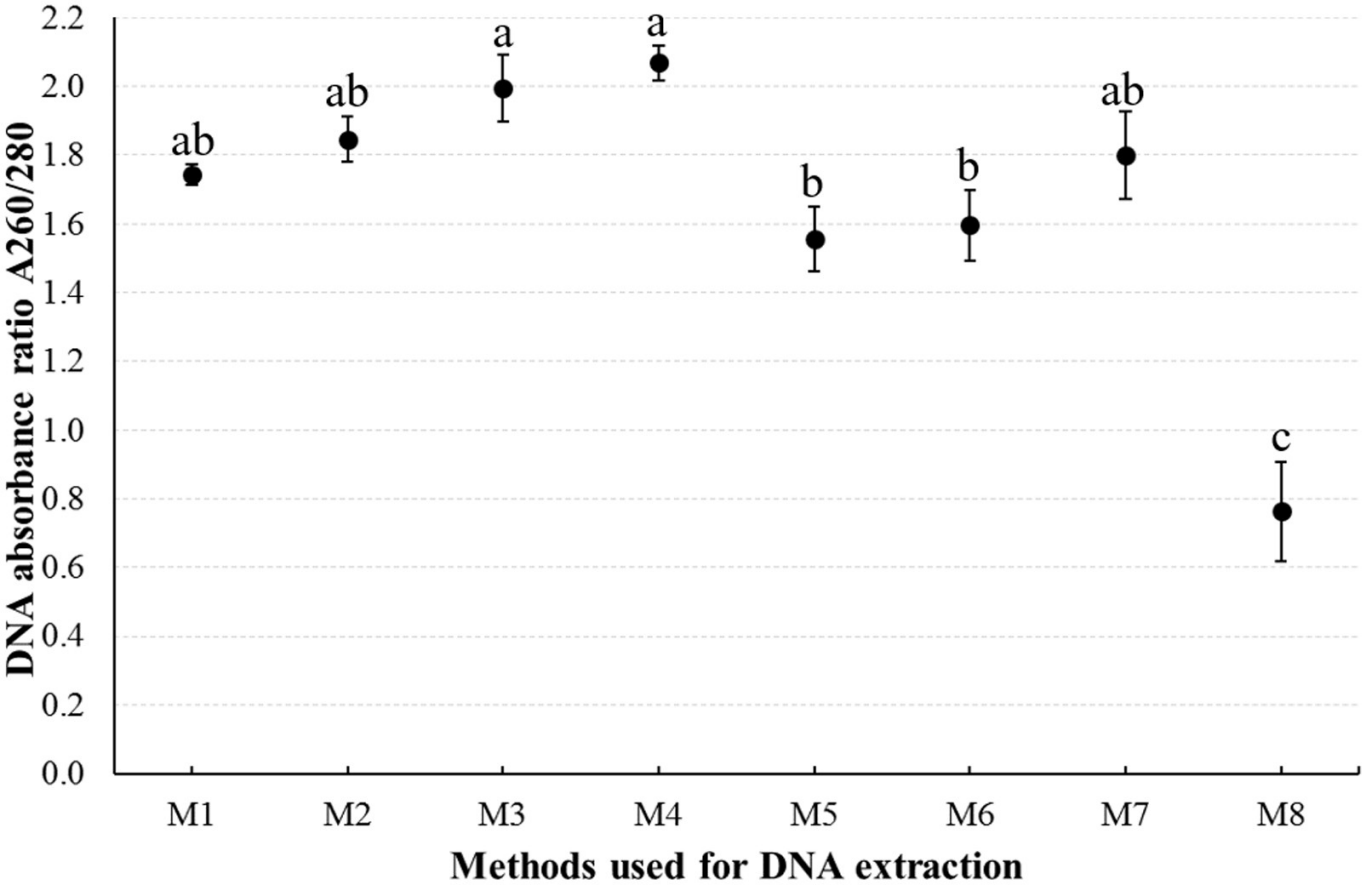
Average absorbance ratio A260/280 (mean±SE) of DNA extracted from fresh and short period conserved samples. Average DNA absorbance ratio A260/280 was calculated based on samples of the 3rd instar female nymph and female adult. Means with different lowercase letters above the bars are significantly different at P < 0.05 (one-way ANOVA, Tukey’s HSD test).

The DNA extracted from the fresh and short period preserved samples was generally high quality. That the absorbance ratios (S2 Table) of extracted DNA from specimens preserved for intermediate and long periods (*i*.*e*., Preservation status III and IV) (Table 1) were generally greater than (or even unmeasurable) that of the fresh and short period preserved samples might be because of DNA degradation after longer storage [26]. Mean absorbance ratios of the DNA extracted using the methods M1, M2, and M7 were about 1.80 (Fig 3). Although the DNA yield rate was low with methods M1 and M2 (P < 0.05, Table 2), the quality (average absorbance ratios were more closer to 1.80) of the DNA extracted by using these methods, which both use RNase A to remove contaminant RNA, was the highest among the eight methods (Fig 3). For method M7, the DNA absorbance ratio had great variation in value (Fig 3). Mean ratios for DNA extracted using the methods M3 and M4 were higher than 1.80 (Fig 3), indicating that the DNA might be degraded or contaminated by RNA [26]. Ratios for DNA extracted by methods M5 and M6 were lower than 1.80 (Fig 3), showing that the DNA could be contaminated by protein [26]. The DNA extracted using the M8 method was light yellow in color, making it impossible to authentically determine the amount of extracted DNA or impurities (Table 2). Statistically, the absorbance ratio was significantly affected by the eight different DNA extraction methods (F = 23.87, df = 7, P < 0.01). The average absorbance ratio of M8 was lower than those of the seven other methods, and those of M3 and M4 methods were higher than M5 and M6 methods (P < 0.05) (Fig 3).

**Table 2:** DNA yield extracted by using eight commonly used methods from the fresh and short period preserved samples.

No.	Methods	Abbreviated as	DNA yield rate (ng·mg^-1^) (mean±SE) ^a^
M1	Sodium chloride method	NaCl	1422±124 f
M2	SDS-RNase A method	SDSR	1898±155 e
M3	Sodium dodecyl sulfate method	SDS	8589±831 bc
M4	DNeasy Blood & Tissue kit	DNeasy	4138±501 d
M5	Chloroform-isopentyl alcohol method	Chloroform	9757±1059 b
M6	Acetic acid potassium method	KAc	12718±1378 a
M7	Salting-out method	Salt	7066±240 c
M8	Rapid method	Rapid	Null

Means followed by different lowercase letters are significantly different at P < 0.05 (one-way ANOVA, Tukey’s HSD test).

^a^ DNA yield was calculated based on DNA concentration, DNA volume, and body mass of the 3rd instar nymph and female adult of the fresh and short period preserved samples.

The DNA extraction method also had a significant effect on DNA yield rate (F = 104.53, df = 6, P < 0.01). The yield rates by the M6, M5, M3, and M7 methods were significantly higher than that obtained by the M4 method, while those by the M2 and M1 methods were significantly lower (P < 0.05, Table 2).

#### Time and cost consumed

We calculated the estimated time in hours and cost in US dollars (USD) for each method used to extract DNA from an individual specimen (Table 3). The time period for each of the eight DNA extraction methods ranged from 0.7 to 7.1 h (Table 3). M1 was the most time-consuming method, followed by M4 and M6, and then by M2 and M5. M8, a crude DNA extraction method with no need for further precipitation or dissolution of the DNA, was the most time-saving approach. M3 and M7 were relatively time-saving compared to the other five methods (Table 3).

**Table 3:** Estimated cost and time required per extraction by using eight commonly used methods.

No.	Methods	Estimated time per extraction (h)	Estimated cost per sample (USD)	Comprehensive time and cost ^a^
M1	NaCl	7.1	0.440	0.126
M2	SDSR	3.2	0.449	0.058
M3	SDS	2.2	0.228	0.020
M4	DNeasy	4.2	3.482	0.592
M5	Chloroform	2.9	0.215	0.025
M6	KAc	3.9	0.214	0.034
M7	Salt	2.2	0.214	0.019
M8	Rapid	0.7	0.055	0.002

^a^ Comprehensive time and cost were calculated as: (estimated cost per extraction of any one method / maximum estimated cost among eight methods) × (estimated time per extraction of any one method / maximum estimated time among eight methods).

M4 was the most expensive among the eight methods, followed by the methods M2, M1, and then M3, M5, M6, and M7. Method M4 used a silica-based membrane to bind DNA and was relatively simpler compared to the other six DNA precipitation methods. M8 was the most cost-saving approach (Table 3). Because laboratory prepared DNA extraction buffers and other reagents are low cost, all seven other methods were much cheaper than was DNeasy (M4). Use of RNase A to remove contaminant RNA almost doubled the cost of methods M1 and M2 when compared to methods M3, M5, M6, and M7 (Table 3).

When time and cost were considered together, M8 was the most economical among the eight methods, followed by M7, M3, and M5. Among the least economical were M6 and M2, while M4 consumed the most combined time and cost.

### Comprehensive analysis

When DNA quality and quantity obtained, the cost and time required, and overall PCR performance were considered, the SDS (M3) method was generalized as the most suitable for DNA extraction from mealybugs preserved for different time periods. Results from this method were comparable with those obtained from the commercial DNeasy kit (M4). This method resulted in higher PCR performance (Fig 2), DNA yield (Table 2) and quality (Figs 1 and 3) with lesser time and cost needed (Table 3). The Rapid (M8) method was the most time- and cost- saving method (Table 3) and worked especially well for egg and 1st instar nymph samples (Fig 2).

### Application of DNA extracted with the SDS method

To investigate the quantity of available DNA extracted from an individual mealybug by using method M3 for PCR product sequencing, PCR amplifications of COI and 28S genes were tested on 2-fold serial dilutions of DNA from individual fresh female adult samples.

**Fig 4:**
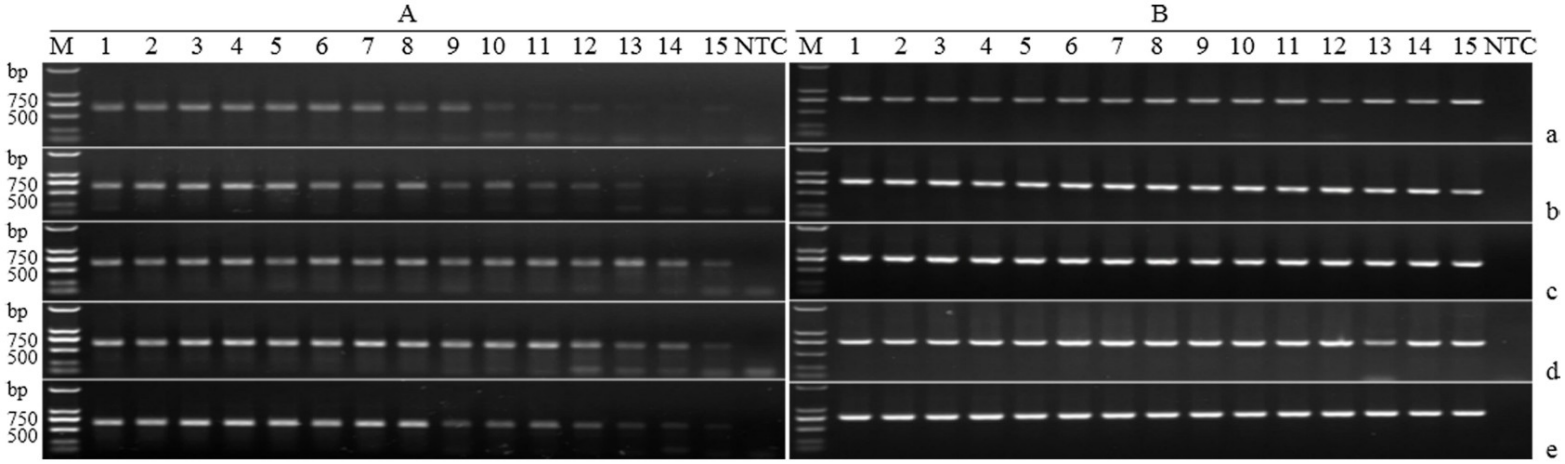
PCR amplification of the COI (A) and 28S (B) genes using a DNA template dilution series of fresh samples extracted using the SDS method. Five individuals of female adults are a-e. Lanes 1–15: 166.20×10^3^, 83.10×10^3^, 41.55×10^3^, 20.78×10^3^, 10.39×10^3^, 5.19×10^3^, 2.60×10^3^, 1.30×10^3^, 649.22, 324.61, 162.30, 81.15, 40.58, 20.29, 10.14 pg·μL^-1^, *i*.*e*., 1-, 2-, 4-, 8-, 16-, 32-, 64-, 128-, 256-, 512-, 1,024-, 2,048-, 4,096-, 8,192-, and 16,384-fold dilutions, respectively. M: Trans2K DNA Marker; NTC: negative control (ultrapure water).

Sufficient DNA was retrieved to enable PCR amplification and sequencing of COI gene at a concentration of 324.61 pg·μL^-1^ (a 512-fold dilution, equal to 1/13,653 of the DNA of a female adult was used) (Fig 4A, lane 10). In addition, the PCR products were recovered and sequenced successfully for 2 of 5 samples at a DNA concentration of 10.14 pg·μL^-1^ (a 16,384-fold dilution, equal to 1/436,907 of the DNA of a female adult was used) (Fig 4A, lane 15). For the 28S gene, PCR amplification and sequencing of all PCR products with all DNA dilutions resulted in sequences of good quality even at 16,384-fold dilution (Fig 4B, lane 15). This indicated that the DNA extracted from an individual mealybug by using the M3 method could provide enough DNA for more than 10,000 PCR. Furthermore, BLAST analysis revealed 100% identity between the sequences of the COI or 28S genes generated in this study and those in GenBank.

## Discussion

The critical first step for most molecular research is DNA extraction [15,16], which should result in sufficient DNA of high enough quality for subsequent experiments. The extraction method should also minimize investment of time, effort, and cost [14,18]. Efficacy of methods used can depend on specimen identity and preservation [12,18,22]. In the current study, we clarified which commonly used DNA extraction methods perform best for mealybug samples across life stages and time preserved.

Both extraction method and preservation time played a role in determining DNA quality and quantity obtained from mealybug samples. Samples that were fresh or preserved for only a short period (Preservation status I and II) resulted in higher DNA yield and quality (Figs 1 and 3, Table 2). The NaCl (M1), SDSR (M2), SDS (M3), and DNeasy (M4) methods were effective at isolating adequate amounts of quality DNA (Figs 1 and 3, Table 2), based on the absorbance ratio (Fig 3) and the presence of a ~23 kb main band (Fig 1). These methods can thus be employed for amplification of long fragment genes [44]. Other methods showed variable levels of contamination. KAc (M6) and Chloroform (M5) showed a relatively high protein contamination, as indicated by the absorbance ratio (Fig 3). Results from the SDS (M3) and Salt (M7) methods had smear tails present, indications of RNA contamination or DNA degradation (Fig 1A and 1B). Methods employing RNase A (NaCl (M1) and SDSR (M2)) effectively removed the RNA contamination (Fig 3). A study by Shi *et al*. [35] had similar findings: the NaCl method with RNase A was more effective than the SDS method, which uses β-mercaptoethanol, an RNase A inhibitor [45]. When the SDS method (M3) was modified to incorporate RNase A (SDSR method (M2)), RNA contamination was eliminated, and the SDSR method resulted in more DNA than did the NaCl method (M1) (Figs 1 and 3, Table 2). Absorbance ratios for the DNeasy method (M4) were higher than 1.80 (Fig 3), and no RNA contamination was detected (Fig 1). However, RNA contamination in the samples in the current study was so low as to have no detectable effect on PCR amplification (Fig 2) or other downstream applications [45].

The small body size of mealybugs from early life stages (eggs and 1st and 2nd instar nymphs) made the amount and concentration of DNA extracted from these samples impossible to measure with the spectrophotometer [12]. To address this limitation, we converted the DNA yield from later ontogenetic stages (3rd instar female nymphs and female adults) into a DNA yield rate [18]. It was assumed that the samples of closely related mealybugs species contained a consistent DNA state (S5 Table) [21]. We also ignored the difference of DNA rate between 3rd instar female nymph and female adult (S5 Table) [18], because they were similar [46]. Samples that were fresh or preserved for a short period could be effectively extracted by the methods of NaCl (M1), SDSR (M2), SDS (M3), and DNeasy (M4), which generated yield rates ranging from 1,422 ng·mg^-1^ to 8,589 ng·mg^-1^ (Table 2). Our results are in alignment with those of Chen *et al*. [18], which found that the SDS method was significantly more effective than the DNeasy kit.

Amount and degradation of DNA extracted from insect specimens depend on the preservation time and conditions [18]. DNA degradation can be hastened by the presence of free water, oxygen, UV radiation, and higher temperatures [22,23,25]. Vink *et al*. [21] found that degradation occurred more rapidly when samples were stored above -20 °C or in ethanol for more than 6 wks. DNA degradation intensifies with lengthening time period [22]. In the current study, DNA extracted from specimens stored for 72 or 137 wks showed smears towards the low molecular weight portion of the gel (Fig 1C and 1D) and greater absorbance ratios (or unmeasurable) were detected than that of the fresh and short period preserved samples (S2 Table), indicating that severe DNA degradation had happened [26]. Despite these effects, DNA extracted from beetle samples stored dry for 25 yrs could still be used for PCR amplification with the SDS method [25].

The initial amount of target DNA determines the success of a PCR reaction, and is thus dependent on the rate of degradation [12,18,26]. In even the samples stored for longer time periods, DNA fragments were successfully amplified for genes with high copy numbers (*i*.*e*., mtDNA COI) but also those with low numbers (*i*.*e*., 28S rDNA) [23] (Fig 2). In small degraded samples, like eggs stored for long periods, a weak band was detected for the DNA samples (Fig 2), however, a bright main band was observed when more egg DNA template was added (personal observation). These findings are consistent with those of Wei *et al*. [47], which showed that DNA from long term (570 d, ≈81 wks) preserved mealybugs could be used in PCR reactions. The method used also determined how effective DNA extraction and amplification could be. Amplification with the KAc (M6) method was extremely low (Fig 2A). Despite high protein contamination with the Chloroform (M5) method or unstable quality with the Salt (M7) method (Fig 3), these methods still resulted in satisfactory amplification of the COI (Fig 2A) and 28S genes (Fig 2B). Although the PCR amplification could be influenced by the unnormalized DNA concentrations of different DNA extraction methods, generally, the NaCl (M1), SDSR (M2), SDS (M3), Chloroform (M5), and Salt (M7) methods were all equal to or more successful than the DNeasy kit (M4) for PCR (Fig 2). Previous work has found that the high copy numbers of mtDNA allow for increased PCR amplification over the low copy numbers of rDNA [23]. Our study found, however, that some samples yielded amplifiable 28S rDNA but failed in amplification of mtDNA COI gene (Fig 2). Additionally, all eight methods evaluated by our study were approximately equal in ability to yield PCR products of the 28S gene (Fig 2B). This phenomenon may be a result of sequence divergence at the primer binding sites [25].

In terms of time and monetary effectiveness, the Rapid (M8) method outperformed the others evaluated in this study (Table 3). This method is the most straightforward with regard to laboratory supplies and methods: DNA extraction occurs in a single tube and the crude extracts can be directly used for subsequent steps [40]. DNA extraction was effective with even egg samples and yielded PCR products sufficient for sequencing (Fig 2). However, DNA from later life stages was somewhat yellow in color. DNA extraction kits, including DNeasy, can be expensive compared to traditional extraction methods [14]. The other seven methods evaluated in this study averaged less than USD $0.45 per sample extraction, or approximately 13% of procedures conducted with the DNeasy kit (Table 3). Use of a single reagent, *e*.*g*., RNase A, can nearly double the cost of an otherwise inexpensive extraction method (Table 3). Usually, DNA extraction mainly relies on the digestive function of enzymes, *e*.*g*., proteinase K used for cell lysis [22]. Digestion time for the eight methods ranged from 0.5 to 5 h, with the Rapid method (M8) the most effective, followed by SDS (M3) and Salt (M7). Incubation with RNase A added substantial time to methods that included it (NaCl (M1) and SDSR (M2)). Methods NaCl (M1) and DNeasy (M4) incorporate 4–5 h for incubation of proteinase K, which are relatively time-consuming. The DNeasy kit (M4) lives up to its name in some regards, and is simple and effective and labor-saving than the other six methods, besides the Rapid method (M8).

## Conclusions

Comprehensive analysis of DNA yield and quality, expenses of cost and time, and PCR amplification and sequencing performance indicated that the SDS method (M3) was the most effective, outperforming even standard commonly-used kits (*e*.*g*., DNeasy; M4). Using this method, the DNA extracted from even a single adult female mealybug could be used for more than 10,000 PCR replicates. The SDS method was further recommended by its sufficient performance across life stages (2nd and 3rd instar nymphs, and adult) and preservation durations (including fresh samples, and short, intermediate, and long-term preservation). For very early stages (eggs and 1st instar nymphs), the Rapid method (M8) proved the most effective. Our findings can help to guide methods of detection and analysis for studies of invasive mealybugs and other small insects based on life stage and preservation status.

## Supporting information

S1 File Detailed procedure of the eight commonly used DNA extraction methods. (DOC)

S1 Table Concentration of DNA (ng·μL^-1^) extracted by using eight commonly used methods from mealybug specimens preserved at different time periods. (DOC)

S2 Table Absorbance ratio A260/280 of DNA extracted by using eight commonly used methods from mealybug specimens preserved at different time periods. (DOC)

S3 Table Individual body size (length×width, mm) of mealybug samples. (DOC)

S4 Table Estimated individual body mass (mg) of mealybug samples. (DOC)

S5 Table Yield rate of DNA (ng·mg^-1^) extracted by using eight commonly used methods from mealybug specimens fresh and preserved at short time period. (DOC)

S1 Data (ZIP)
