# Supplementary material for: Comparative analysis of eight DNA extraction methods for molecular research in mealybugs
Source: PLoS One. 2019 Dec 31;14(12):e0226818. doi: 10.1371/journal.pone.0226818 (PMC6938366; doi:10.1371/journal.pone.0226818)
Supplement: S1 File — (DOC) [file pone.0226818.s001.doc]

**S1 File. Detailed procedure of the eight commonly used DNA extraction methods.**

Method 1 (M1), the sodium chloride method (NaCl), as described by Shi *et al*. [1]. Step 1: Mealybugs were individually homogenized on a piece of parafilm (Bemis, Neenah, WI, USA) using a sterile PCR tube with 20 μL DNA extraction buffer (10 mM Tris-HCl, 100 mM NaCl, 1 mM EDTA, 0.75% SDS, pH 8.0). The homogenate was placed in a sterile 1.5 mL centrifuge tube. The homogenizer (parafilm and the PCR tube) was washed with 280 μL DNA extraction buffer, then transferred to the same tube.

Step 2: Add 5 μL of RNase A (10 mg·mL^-1^) into the tube, vortex briefly, incubate at 37 ºC for 1 h. Mix occasionally during the incubation.

Step 3: Add 5 μL of proteinase K (20 mg·mL^-1^) into the tube, vortex briefly, incubate at 56 ºC for 4 h. Mix occasionally during incubation.

Step 4: Add 300 μL of ice-cold NaCl (5 M), and mix thoroughly. Centrifuge for 30 min at 10,000 r·min^-1^.

Step 5: Pipet the supernatant to a new 1.5 mL centrifuge tube, mix with one volume of ice-cold isopropanol. Place the mixture at -20 ºC for 1 h. Centrifuge for 15 min at 12,000 r·min^-1^. Discard the supernatant.

Step 6: Wash the DNA pellet with ice-cold 75% ethanol. Centrifuge for 10 min at 10,000 r·min^-1^. Discard the supernatant.

Step 7: Air-dry. Add 40 μL ultrapure water (20 μL for egg; 30 μL for 1st and 2nd instar nymphs) to dissolve DNA.

Method 2 (M2), the sodium dodecyl sulfate (SDS)-RNase A method (SDSR), as described by Phillips & Simon [2].

Step 1: Mealybugs were individually homogenized on a piece of parafilm (Bemis, Neenah, WI, USA) using a sterile PCR tube with 20 μL DNA extraction buffer (100 mM NaCl, 10 mM Tris-HCl, 50 mM EDTA, 0.5% SDS, 0.2% β-mercaptoethanol, pH 8.0). The homogenate was placed in a sterile 1.5 mL centrifuge tube. The homogenizer (parafilm and PCR tube) was washed with 180 μL DNA extraction buffer, then transferred to the same tube.

Step 2: Add 5 μL of RNase A (10 mg·mL^-1^) into the tube, vortex briefly, incubate at 37 ºC for 1 h. Mix occasionally during the incubation.

Step 3: Add 5 μL of proteinase K (20 mg·mL^-1^) into the tube, vortex briefly, incubate at 65 ºC for 40 min. Mix occasionally during the incubation.

Step 4: Centrifuge for 10 min at 7,500 r·min^-1^. Pipet the supernatant to a new 1.5 mL centrifuge tube.

Step 5: Add 150 μL of ice-cold NaCl (5 M), and mix thoroughly. Centrifuge for 15 min at 12,000 r·min^-1^.

Step 6: Pipet the supernatant to a new 1.5 mL centrifuge tube, mix with one volume of ice-cold isopropanol. Place the mixture at -20 ºC for 30 min. Centrifuge for 15 min at 12,000 r·min^-1^. Discard the supernatant.

Step 7: Wash the DNA pellet with ice-cold 75% ethanol. Centrifuge for 10 min at 10,000 r·min^-1^. Discard the supernatant.

Step 8: Air-dry. Add 40 μL ultrapure water (20 μL for egg; 30 μL for 1st and 2nd instar nymphs) to dissolve DNA.

Method 3 (M3), the sodium dodecyl sulfate method (SDS), as described by Phillips & Simon [2].

Step 1: Mealybugs were individually homogenized on a piece of parafilm (Bemis, Neenah, WI, USA) using a sterile PCR tube with 20 μL DNA extraction buffer (100 mM NaCl, 10 mM Tris-HCl, 50 mM EDTA, 0.5% SDS, 0.2% β-mercaptoethanol, pH 8.0). The homogenate was placed in a sterile 1.5 mL centrifuge tube. The homogenizer (parafilm and the PCR tube) was washed with 180 μL DNA extraction buffer, and then transferred to the same tube.

Step 2: Add 5 μL of proteinase K (20 mg·mL^-1^) into the tube, vortex briefly, incubate at 65 ºC for 40 min, and mix occasionally during the incubation.

Step 3: Centrifuge for 10 min at 7,500 r·min^-1^. Pipet the supernatant to a new 1.5 mL centrifuge tube.

Step 4: Add 150 μL of ice-cold NaCl (5 M), and mix thoroughly. Centrifuge for 15 min at 12,000 r·min^-1^.

Step 5: Pipet the supernatant to a new 1.5 mL centrifuge tube, mix with one volume of ice-cold isopropanol. Place the mixture at -20 ºC for 30 min. Centrifuge for 15 min at 12,000 r·min^-1^. Discard the supernatant.

Step 6: Wash the DNA pellet with ice-cold 75% ethanol. Centrifuge for 10 min at 10,000 r·min^-1^. Discard the supernatant.

Step 7: Air-dry. Add 40 μL ultrapure water (20 μL for egg; 30 μL for 1st and 2nd instar nymphs) to dissolve the DNA.

Method 4 (M4), DNeasy Blood & Tissue kit (DNeasy) (Qiagen, Duesseldorf, Germany), following the instructions of the manufacturer.

Step 1: Mealybugs were individually homogenized on a piece of parafilm (Bemis, Neenah, WI, USA) using a sterile PCR tube with 20 μL buffer ATL. The homogenate was placed in a sterile 1.5 mL centrifuge tube. The homogenizer (parafilm and the PCR tube) was washed with 160 μL buffer ATL, then transferred to the same centrifuge tube.

Step 2: Add 20 μL proteinase K. Mix thoroughly by vortexing, and incubate at 56 °C for 4 h. Mix occasionally during the incubation.

Step 3: Vortex for 15 s. Add 200 μL buffer AL, and mix thoroughly by vortexing. Then add 200 μL 99.7% ethanol, and vortex again.

Step 4: Pipet the mixture from the step 3 into a DNeasy Mini spin column sited in the 2 mL collection tube. Centrifuge at 8,000 rpm for 1 min. Discard the collection tube and flow-through.

Step 5: Place the DNeasy Mini spin column in a new 2 mL collection tube, add 500 μL buffer AW1, and centrifuge for 1 min at 8,000 rpm. Discard the collection tube and flow-through.

Step 6: Place the DNeasy Mini spin column in a new 2 mL collection tube, add 500 μL buffer AW2, and centrifuge for 3 min at 14,000 rpm. Discard the collection tube and flow-through.

Step 7: Place the DNeasy Mini spin column in a sterile 1.5 mL centrifuge tube, and pipet 40 μL ultrapure water (20 μL for egg; 30 μL for 1st and 2nd instar nymphs) onto the DNeasy membrane.

Step 8: Incubate at room temperature for 1 min, and then centrifuge for 1 min at 8,000 rpm.

Method 5 (M5), the chloroform-isopentyl alcohol method (Chloroform), as described by Zhou *et al*. [3].

Step 1: Mealybugs were individually homogenized on a piece of parafilm (Bemis, Neenah, WI, USA) using a sterile PCR tube with 20 μL DNA extraction buffer (50 mM Tris-HCl, l mM EDTA, 1% SDS, 20 mM NaCl, pH 8.0). The homogenate was placed in a sterile 1.5 mL centrifuge tube. The homogenizer (parafilm and the PCR tube) was washed with 180 μL DNA extraction buffer, then transferred to the same tube.

Step 2: Add 5 μL of proteinase K (20 mg·mL^-1^) into the tube, vortex briefly, incubate at 60 ºC for 1 h. Mix occasionally during the incubation. Then boiled for 5 min.

Step 3: Add 200 μL of ice-cold chloroform/isopentyl alcohol (24:1), and mix briefly. Keep the mixture on ice for 30 min. Centrifuge for 10 min at 10,000 r·min^-1^.

Step 5: Pipet the supernatant to a new 1.5 mL centrifuge tube, mix with two volume of ice-cold 99.7% ethanol. Place the mixture at -20 ºC for 30 min. Centrifuge for 15 min at 10,000 r·min^-1^. Discard the supernatant.

Step 6: Wash the DNA pellet with ice-cold 75% ethanol. Centrifuge for 15 min at 10,000 r·min^-1^. Discard the supernatant.

Step 7: Air-dry. Add 40 μL ultrapure water (20 μL for egg; 30 μL for 1st and 2nd instar nymphs) to dissolve DNA.

Method 6 (M6), the acetic acid potassium method (KAc), as described by Dai *et al*. [4].

Step 1: Mealybugs were individually homogenized on a piece of parafilm (Bemis, Neenah, WI, USA) using a sterile PCR tube with 20 μL DNA extraction buffer (50 mM Tris-HCl, 25 mM NaCl, 25mM EDTA, 0.5% SDS, pH 8.0). The homogenate was placed in a sterile 1.5 mL centrifuge tube. The homogenizer (parafilm and the PCR tube) was washed with 180 μL DNA extraction buffer, then transferred to the same tube.

Step 2: Add 5 μL of proteinase K (20 mg·mL^-1^) into the tube, vortex briefly, incubate at 65 ºC for 1 h. Mix occasionally during the incubation.

Step 3: Add 200 μL of ice-cold KAc (3 M), and mix thoroughly. Keep the mixture on ice for 1 h. Centrifuge for 10 min at 12,000 r·min^-1^.

Step 4: Pipet the supernatant to a new 1.5 mL centrifuge tube, mix with two volume of ice-cold 99.7% ethanol. Place the mixture at -20 ºC for 1 h. Centrifuge for 20 min at 12,000 r·min^-1^. Discard the supernatant.

Step 5: Wash the DNA pellet with ice-cold 75% ethanol. Centrifuge for 15 min at 10,000 r·min^-1^. Discard the supernatant.

Step 6: Air-dry. Add 40 μL ultrapure water (20 μL for egg; 30 μL for 1st and 2nd instar nymphs) to dissolve the DNA.

Method 7 (M7), the salting-out method (Salt), as described by Sunnucks & Hales [5].

Step 1: Mealybugs were individually homogenized on a piece of parafilm (Bemis, Neenah, WI, USA) using a sterile PCR tube with 20 μL DNA extraction buffer (50 mM Tris-HCl, 400 mM NaCl, 20 mM EDTA, 0.5% SDS, pH 7.5). The homogenate was placed in a sterile 1.5 mL centrifuge tube. The homogenizer (parafilm and the PCR tube) was washed with 280 μL DNA extraction buffer, then transferred to the same tube.

Step 2: Add 5 μL of proteinase K (20 mg·mL^-1^) into the tube, vortex briefly, incubate at 60 ºC for 1 h. Mix occasionally during the incubation.

Step 3: Add 80 μL of ice-cold NaCl (5 M), and mix thoroughly. Centrifuge for 10 min at 14,000 r·min^-1^.

Step 5: Pipet the supernatant to a new 1.5 mL centrifuge tube, mix with two volume of ice-cold 99.7% ethanol. Place the mixture at -20 ºC for 30 min. Centrifuge for 10 min at 14,000 r·min^-1^. Discard the supernatant.

Step 6: Wash the DNA pellet with ice-cold 75% ethanol. Centrifuge for 10 min at 10,000 r·min^-1^. Discard the supernatant.

Step7: Air-dry. Add 40 μL ultrapure water (20 μL for egg; 30 μL for 1st and 2nd instar nymphs) to dissolve DNA.

Method 8 (M8), the rapid method (Rapid), as described by De Barro & Driver [6].

Step 1: Mealybugs were individually homogenized on a piece of parafilm (Bemis, Neenah, WI, USA) using a sterile PCR tube with 20 μL (10 μL for egg, 15 μL for 1st and 2nd instar nymphs) lysis buffer (50 mM Tris-HCl, 50 mM KCl, 0.45% Tween 20, 0.45% NP40, 0.2% gelatin, 80 ug·mL^-1^ proteinase K). The homogenate was placed in a sterile PCR tube. The homogenizer (parafilm and the PCR tube) was washed with 20 μL (10 μL for egg, 15 μL for 1st and 2nd instar nymphs) lysis buffer, then transferred to the same tube.

Step 2: Incubate at 65 °C for 30 min. Boil for 10 min to inactivate proteinase K.

**References**

1. Shi J, Xie YP, Xue JL, Yao GQ. Comparative study of methods for isolation of genomic DNA of scale insects. Chinese Bull Entomol. 2005;42: 207-211.
2. Phillips AJ, Simon C. Simple, efficient, and nondestructive DNA extraction protocol for arthropods. Ann Entomol Soc Am. 1995;88: 281-283.
3. Zhou ZX, Wan FH, Zhang GF, Chen B. A rapid method for extraction of genomic DNA of *Bemisia tabac*i. Plant Protect. 2007;33: 131-133.
4. Dai TM, Lü ZC, Wan FH. Comparison of four methods for whole genomic DNA extraction from *Bemisia tabaci*. Biotech Bull. 2014;8: 70-75.
5. Sunnucks P, Hales DF. Numerous transposed sequences of mitochondrial cytochrome oxidase I-II in Aphids of the genus Sitobion (Hemiptera: Aphididae). Mol Biol Evol. 1996;13: 510-524.
6. De Barro PJ, Driver F. Use of RAPD PCR to distinguish the B biotype from other biotypes of *Bemisia tabaci* (Gennadius) (Hemiptera: Aleyrodidae). Aust J Entomol. 1997;36: 149-152.
