# Supplementary material for: Comparative analysis of eight DNA extraction methods for molecular research in mealybugs
Source: PLoS One. 2019 Dec 31;14(12):e0226818. doi: 10.1371/journal.pone.0226818 (PMC6938366; doi:10.1371/journal.pone.0226818)
Supplement: S1 Table — (DOC) [file pone.0226818.s002.doc]

**S1 Table. Concentration of DNA (ng·μL^-1^) extracted by using eight commonly used methods from mealybug specimens preserved at different time periods.**

| **No.** | **Methods** | **3rd instar nymph** | | **Female adult** | | **3rd instar nymph** | | **Female adult** | |
| --- | --- | --- | --- | --- | --- | --- | --- | --- | --- |
|  |  | **Fresh** | **Short period** | **Fresh** | **Short period** | **Intermediate period** | **Long period** | **Intermediate period** | **Long period** |
| M1 | NaCl | 6.96 | 12.90 | 23.44 | 29.27 | Null | Null | 18.90 | 56.20 |
| M2 | SDSR | 10.90 | 16.40 | 26.80 | 40.80 | 5.47 | 14.90 | 34.30 | Null |
| M3 | SDS | 53.73 | 59.61 | 168.00 | 150.23 | Null | 29.30 | Null | 227.00 |
| M4 | DNeasy | 28.90 | 32.30 | 65.13 | 70.10 | 9.94 | Null | 34.80 | Null |
| M5 | Chloroform | 65.00 | 80.60 | 135.90 | 187.33 | 48.20 | Null | Null | Null |
| M6 | KAc | 81.00 | 110.57 | 175.97 | 244.27 | 85.00 | 38.80 | Null | Null |
| M7 | Salt | 42.50 | 51.43 | 116.00 | 151.87 | Null | 26.80 | 48.20 | 75.70 |
| M8 | Rapid | Null | Null | Null | Null | Null | Null | Null | Null |
