# Supplementary material for: Comparative analysis of eight DNA extraction methods for molecular research in mealybugs
Source: PLoS One. 2019 Dec 31;14(12):e0226818. doi: 10.1371/journal.pone.0226818 (PMC6938366; doi:10.1371/journal.pone.0226818)
Supplement: S2 Table — (DOC) [file pone.0226818.s003.doc]

**S2 Table. Absorbance ratio A260/280 of DNA extracted by using eight commonly used methods from** **mealybug specimens preserved at different time periods.**

| **No.** | **Methods** | **3rd instar nymph** | | **Female adult** | | **Mean±SE** | **3rd instar nymph** | | **Female adult** | | **Mean±SE^a^** |
| --- | --- | --- | --- | --- | --- | --- | --- | --- | --- | --- | --- |
|  |  | **Fresh** | **Short period** | **Fresh** | **Short period** |  | **Intermediate period** | **Long period** | **Intermediate period** | **Long period** |  |
| M1 | NaCl | 1.75 | 1.73 | 1.81 | 1.69 | 1.74±0.03 | Null | 1.75 | 2.00 | 1.95 | 1.90±0.09 |
| M2 | SDSR | 2.00 | 1.74 | 1.80 | 1.85 | 1.85±0.06 | 2.75 | 2.00 | 1.97 | Null | 2.24±0.31 |
| M3 | SDS | 2.13 | 1.79 | 2.14 | 1.93 | 2.00±0.10 | Null | 1.90 | 2.16 | 2.13 | 2.06±0.10 |
| M4 | DNeasy | 2.13 | 2.03 | 2.15 | 1.96 | 2.07±0.05 | 2.18 | Null | 2.14 | 2.11 | 2.14±0.02 |
| M5 | Chloroform | 1.55 | 1.47 | 1.79 | 1.42 | 1.56±0.09 | 1.80 | 1.70 | 1.78 | 1.70 | 1.76±0.04 |
| M6 | KAc | 1.66 | 1.59 | 1.78 | 1.35 | 1.60±0.10 | 1.82 | 1.56 | Null | 1.71 | 1.70±0.09 |
| M7 | Salt | 2.04 | 1.69 | 1.93 | 1.55 | 1.80±0.13 | Null | 1.94 | 2.02 | 1.99 | 1.98±0.03 |
| M8 | Rapid | 1.02 | 0.45 | 0.89 | 0.69 | 0.76±0.14 | Null | 0.62 | 1.14 | 0.80 | 0.85±0.19 |

a. Means calculated based on valid data.
