# Supplementary material for: Comparative analysis of eight DNA extraction methods for molecular research in mealybugs
Source: PLoS One. 2019 Dec 31;14(12):e0226818. doi: 10.1371/journal.pone.0226818 (PMC6938366; doi:10.1371/journal.pone.0226818)
Supplement: S3 Table — (DOC) [file pone.0226818.s004.doc]

**S3 Table. Individual body size (length×width, mm) of mealybug samples**.

|  | **Length (mm)** | | | | **Width (mm)** | | | |
| --- | --- | --- | --- | --- | --- | --- | --- | --- |
| **Ontogenic stage** | **1** | **2** | **3** | **Mean±SE** | **1** | **2** | **3** | **Mean±SE** |
| 1^st^ instar nymph | 0.5361 | 0.5786 | 0.5684 | 0.5610±0.0157 | 0.2952 | 0.3083 | 0.3315 | 0.3117±0.0130 |
| 2^nd^ instar nymph | 0.9141 | 0.8430 | 0.9425 | 0.8999±0.0362 | 0.4136 | 0.4473 | 0.5012 | 0.4540±0.0312 |
| 3rd instar nymph | 1.4799 | 1.5355 | 1.3930 | 1.4695±0.0508 | 0.7972 | 0.8609 | 0.8391 | 0.8324±0.0229 |
| Female adult | 2.7876 | 3.0119 | 2.9713 | 2.9236±0.0845 | 1.2061 | 1.2989 | 1.2617 | 1.2556±0.0330 |

Take the fresh *phenacoccus solenopsis* samples as an example.
