# Supplementary material for: Comparative analysis of eight DNA extraction methods for molecular research in mealybugs
Source: PLoS One. 2019 Dec 31;14(12):e0226818. doi: 10.1371/journal.pone.0226818 (PMC6938366; doi:10.1371/journal.pone.0226818)
Supplement: S4 Table — (DOC) [file pone.0226818.s005.doc]

**S4 Table. Estimated individual body mass (mg) of mealybug samples**.

| **Ontogenic stage** | **Fresh** ^a^ | | | | **Short period** ^b^ | | | |
| --- | --- | --- | --- | --- | --- | --- | --- | --- |
|  | **1** | **2** | **3** | **Mean±SE** | **1** | **2** | **3** | **Mean±SE** |
| 3rd instar nymph | 0.1800 | 0.2210 | 0.2630 | 0.2213±0.0294 | 0.2967 | 0.2500 | 0.3444 | 0.2970±0.0334 |
| Female adult | 0.6850 | 0.5810 | 0.7900 | 0.6853±0.0739 | 0.8800 | 1.0333 | 0.7333 | 0.8822±0.1061 |

a. Measured in groups of 10 individuals; b. Measured in groups of nine individuals.
