# Supplementary material for: Comparative analysis of eight DNA extraction methods for molecular research in mealybugs
Source: PLoS One. 2019 Dec 31;14(12):e0226818. doi: 10.1371/journal.pone.0226818 (PMC6938366; doi:10.1371/journal.pone.0226818)
Supplement: S5 Table — (DOC) [file pone.0226818.s006.doc]

**S5 Table. Yield rate of DNA (ng·mg^-1^) extracted** **by using eight commonly used methods from mealybug specimens fresh and preserved at short time period.**

| **No.** | **Methods** | **3rd instar nymph** | | **Female adult** | | **Mean±SE** |
| --- | --- | --- | --- | --- | --- | --- |
|  |  | **Fresh** | **Short period** | **Fresh** | **Short period** |  |
| M1 | NaCl | 1257 | 1737 | 1368 | 1327 | 1422±124 |
| M2 | SDSR | 1970 | 2208 | 1564 | 1850 | 1898±155 |
| M3 | SDS | 9711 | 8027 | 9805 | 6812 | 8589±831 |
| M4 | DNeasy | 5223 | 4350 | 3802 | 3178 | 4138±501 |
| M5 | Chloroform | 11747 | 10854 | 7932 | 8494 | 9757±1059 |
| M6 | KAc | 14638 | 14889 | 10270 | 11075 | 12718±1378 |
| M7 | Salt | 7681 | 6926 | 6770 | 6886 | 7066±240 |
| M8 | Rapid | Null | Null | Null | Null | Null |
