# Supplementary figures and images for: Comparative analysis of eight DNA extraction methods for molecular research in mealybugs
Source: PLoS One. 2019 Dec 31;14(12):e0226818. doi: 10.1371/journal.pone.0226818 (PMC6938366; doi:10.1371/journal.pone.0226818)

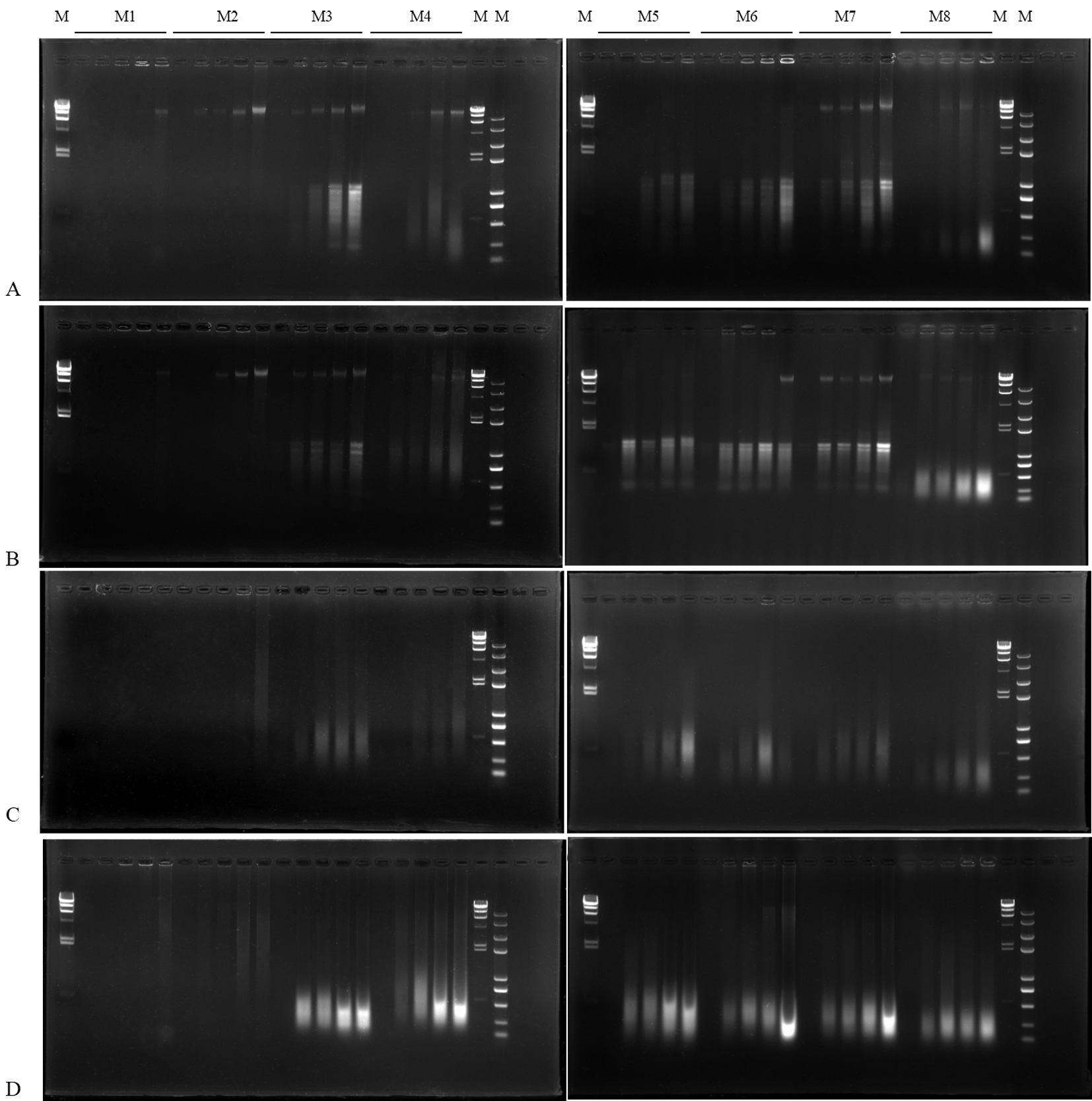

Supplement: S1 Data — (ZIP) [file pone.0226818.s007.zip › Original uncropped gels/Fig 1.tif]

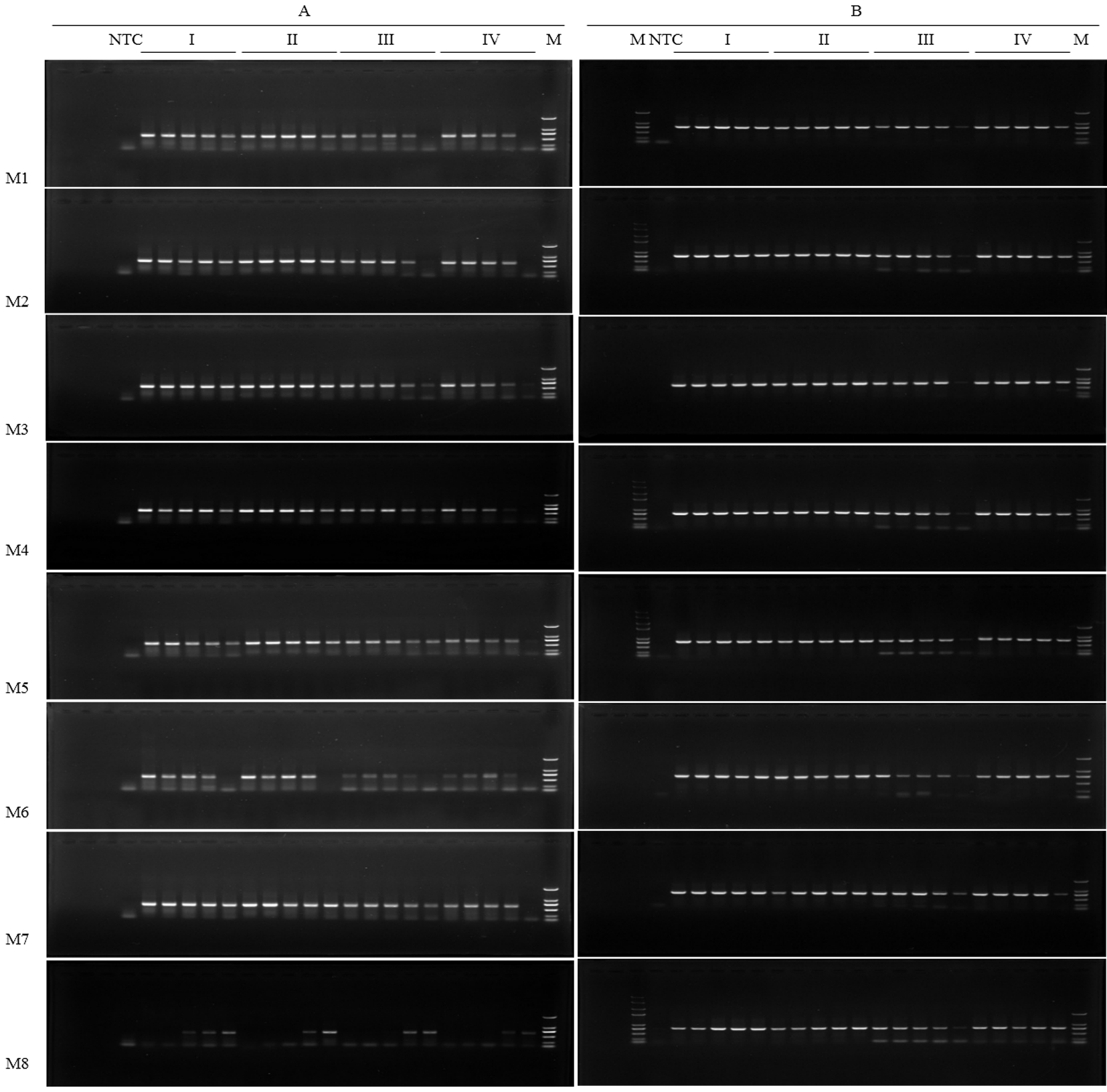

Supplement: S1 Data — (ZIP) [file pone.0226818.s007.zip › Original uncropped gels/Fig 2.tif]

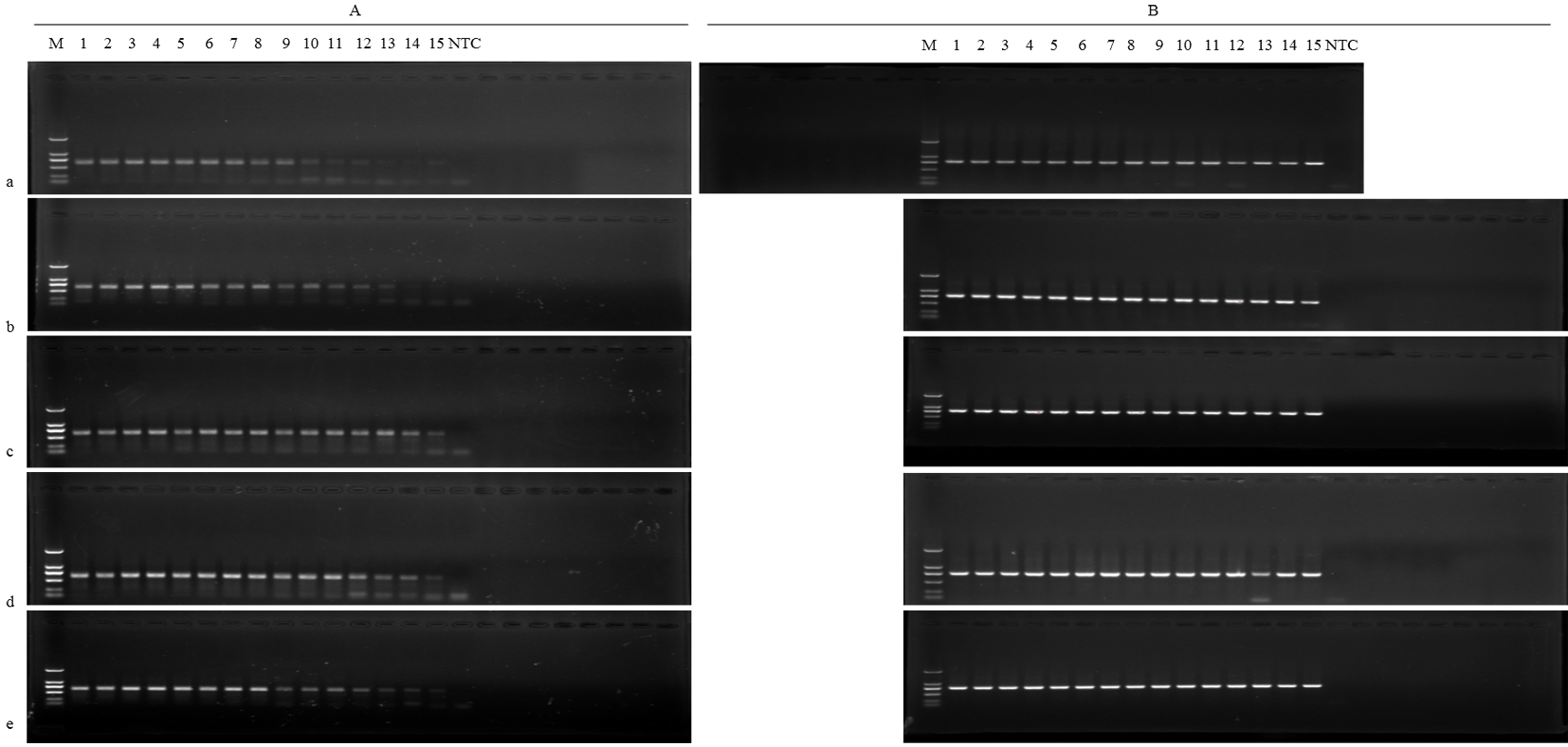

Supplement: S1 Data — (ZIP) [file pone.0226818.s007.zip › Original uncropped gels/Fig 4.tif]
